# Targeting Lung Cancer Stem Cells with Trimebutine: Synergistic Antitumor Activity in Combination with Cisplatin

**DOI:** 10.3390/ijms27188211

**Published:** 2026-09-15

**Authors:** JiHye Seo, Heejin Lee, Dong Kyu Choi, Young-Kyu Kim, Somin Woo, Changkyu Lee, Jeong In Choi, Ge Jiang, Bae Jun Oh, Dong-Seok Lee, Sang-Hyun Min

**Affiliations:** 1 School of Life Sciences & Biotechnology, College of Natural Sciences, Kyungpook National University, Daegu 41566, Republic of Korea; sub04214@naver.com; 2 Daegu ANCHOR Center, Kyungpook National University, Daegu 41566, Republic of Korea; jjunwm@gmail.com (S.W.); leeck30421@knu.ac.kr (C.L.); 3 New Drug Development Center, Daegu-Gyeongbuk Medical Innovation Foundation (K-MEDI Hub), Daegu 41061, Republic of Korea; leeheejin@kmedihub.re.kr (H.L.); kimyk0114@kmedihub.re.kr (Y.-K.K.); baejun.oh@kmedihub.re.kr (B.J.O.); 4 BK21 FOUR KNU Creative BioResearch Group, School of Life Science and Biotechnology, Kyungpook National University, Daegu 41566, Republic of Korea; dongkyu@knu.ac.kr; 5 Graduate School of Pharmacy, Kyungpook National University, Daegu 41566, Republic of Korea; 6 BK21 Infectious Disease Healthcare Program, Kyungpook National University, Daegu 41566, Republic of Korea; 7 Department of Innovative Pharmaceutical Sciences, Kyungpook National University, Daegu 41566, Republic of Korea; 8 Department of Obstetrics and Gynecology, Soonchunhyang University, Bucheon Hospital 170, Jomaru-ro, Bucheon-si 14584, Republic of Korea; rubin@schmc.ac.kr; 9 College of Life and Health, Dalian University, Dalian 116622, China; jiangge1004@163.com

**Keywords:** lung cancer, drug resistance, metastasis, G protein–coupled receptor, anticancer, synergistic effect

## Abstract

Lung cancer recurrence and therapeutic resistance are driven by cancer stem cells (CSCs), yet clinically available CSC-targeted therapies remain lacking. To identify actionable anti-CSC agents, we screened 1018 FDA-approved drugs using a 3D sphere culture system enriching lung CSC-like cells. Trimebutine, a gastrointestinal motility regulator targeting G-protein-coupled receptors (GPCRs), was identified as a potent agent with over 49.5-fold higher selectivity toward lung CSC-like cells (SI > 49.55) compared with standard therapies such as cisplatin (SI = 1.39) and gefitinib (SI = 1.00). In H460-SP spheres, trimebutine markedly impaired sphere-forming capacity, reduced cell viability, and downregulated key CSC surface markers (CD133, CD44) alongside stemness regulators (c-MYC, EpCAM, BMI1, OCT3/4, NANOG, SOX2, SMAD3). Mechanistically, trimebutine suppressed overexpressed calcium and potassium channels (*CACNA1F*, *CACNA1B*, *CACNA1E*, *CACNA1H*, *KCNH2*), attenuating downstream YAP/TAZ–TEAD signaling within the Hippo pathway to trigger apoptosis. In combination with cisplatin, trimebutine exhibited strong synergistic efficacy (Chou–Talalay combination index, CI = 0.015–0.228) in H460-SP spheres and patient-derived lung tumor organoids, suppressing cell viability, stemness marker suppression, and apoptotic cleavage of caspase-3 and PARP compared to monotherapies. Overall, trimebutine targets lung CSC populations by disrupting GPCR–ion channel–Hippo signaling crosstalk, providing a strong preclinical rationale for trimebutine–cisplatin combination strategies to overcome drug resistance and recurrence in lung cancer.

## 1. Introduction

Lung cancer remains the leading cause of cancer-related mortality worldwide [1]. Non-small cell lung cancer (NSCLC) accounts for 80–85% of cases and encompasses adenocarcinoma, squamous cell carcinoma, and large cell carcinoma, whereas small cell lung cancer (SCLC), which is strongly linked to tobacco exposure, represents approximately 15% [2]. A high rate of advanced-stage diagnosis majorly contributes to lung cancer mortality, and even after surgical resection, recurrence is common, with reported recurrence rates of 20–50% [3,4]. Despite the availability of EGFR-targeted therapies, immunotherapies, and cytotoxic chemotherapy, durable responses are limited in many patients [5]. These clinical challenges have prompted efforts to identify the therapeutic vulnerabilities that contribute to recurrence and resistance.

Cancer stem cells (CSCs) are a minority subpopulation of tumor cells that sustain long-term propagation through self-renewal and contribute disproportionately to relapse after therapy [6,7,8]. CSC-like cells have been described in many solid tumors and have been implicated in resistance to cytotoxic therapy and radiotherapy, enabling tumor regrowth following initial tumor debulking [7,9,10,11]. Developmental signaling pathways that regulate normal stem cell self-renewal—including NOTCH, Hippo, Hedgehog, and Wnt/β-catenin—are often co-opted to support CSC-like phenotypes and tumor progression [12,13]. Consequently, the identification of drugs that preferentially suppress CSC-like populations is an active area of translational research.

In this study, we sought to identify clinically approved drugs with preferential activity against lung cancer stem-like cells and to define a rational combination strategy that more effectively suppresses stemness-associated phenotypes. To this end, we first established an optimized three-dimensional (3D) sphere culture system that enriches lung cancer cells with distinct stemness features, thereby providing an in vitro model of lung CSC-like cells. Using this platform, we implemented a 3D sphere–based high-throughput screening approach to interrogate a comprehensive library of Food and Drug Administration (FDA)-approved drugs for their ability to inhibit sphere formation, reduce cell viability, and downregulate core stemness markers. Through this systematic phenotypic screen, we identified trimebutine as a potent agent that robustly suppresses lung CSC-like properties, exhibiting superior CSC-selective efficacy compared with standard first-line lung cancer therapies, including gefitinib and cisplatin. Moreover, we found that combining trimebutine with cisplatin further enhanced anti-tumor activity against lung CSC-like cells, supporting a promising therapeutic strategy to prevent tumor relapse by simultaneously targeting bulk tumor cells and CSC-like subpopulations.

## 2. Results

### 2.1. Trimebutine Preferentially Inhibits the Growth of Lung CSC-like Sphere Culture

First, we enriched cancer stem-like cells (H460-SP) by culturing H460 cells in serum-free medium supplemented with EGF and bFGF on ultra-low attachment (ULA) plates. The successful enrichment of the stem-like population was confirmed by sphere formation capacity and the upregulation of stemness-associated markers compared with parental H460 cells (Supplementary Figure S1a–c).

To identify drug candidates targeting lung cancer stem-like cells, we evaluated a focused panel of 21 candidate compounds prioritized from a primary high-throughput screen of a 1018 FDA-approved compound library [14] based on their sphere-inhibitory activity and safety profiles (Supplementary Table S1). In our multi-step validation workflow, H460-SP cells were cultured in ultra-low attachment 96-well plates and treated with the candidate compounds at 1 and 5 μM. Through ATP-based cell viability assays, candidates that reduced sphere viability by more than 50% compared with the DMSO control were selected (Supplementary Figure S2b). Next, to exclude non-specific cytotoxic agents, counter-screening was performed in parental H460 cells, prioritizing compounds that exhibited minimal toxicity (retaining > 80% viability) (Supplementary Figure S2a). Finally, the selected compounds were evaluated through a sphere formation assay, and those that reduced sphere size by more than 50% compared with the DMSO control were selected (Supplementary Figure S3a,b). Among the candidate hits, trimebutine was ultimately selected for further investigation, as it exhibited potent and selective inhibition against H460-SP spheres without affecting parental H460 cell viability (SI > 45.55).

**Figure 1:**
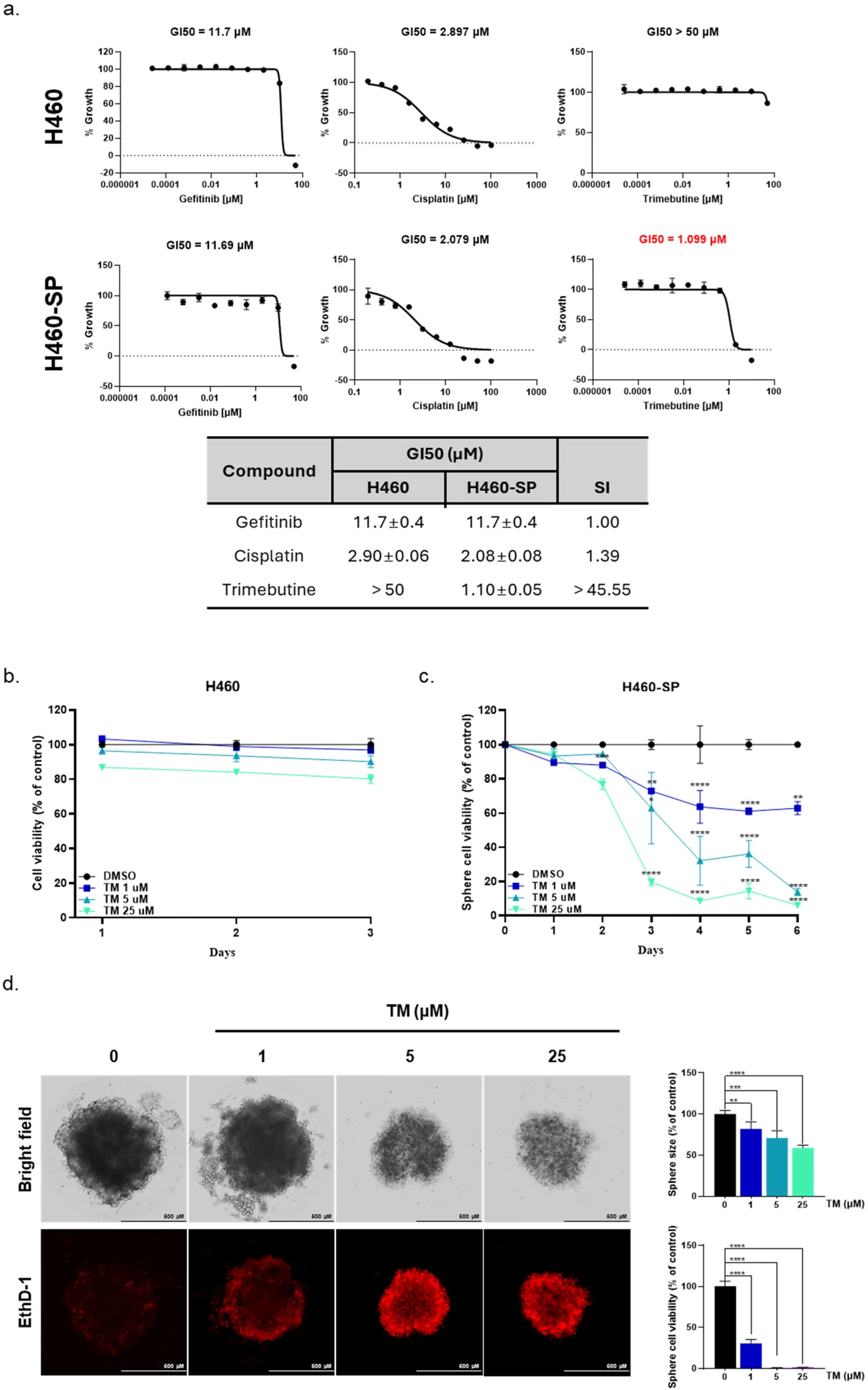
Trimebutine specifically inhibits the growth of lung cancer stem-like cells. (**a**) H460 cells and H460-SP (spheroids) were treated with gefitinib, cisplatin, or trimebutine for 3 or 6 days, respectively, and cell viability was measured using an ATP-based CellTiter-Glo assay. GI50 values and Selectivity indices ($\mathrm{SI}={\mathrm{GI}}_{50,\mathrm{parental}}/{\mathrm{GI}}_{50,\mathrm{sphere}}$) were determined by nonlinear regression analysis using GraphPad Prism 8. (**b**,**c**) Cell viability of H460 (3 days) and H460-SP (6 days) cells following treatment was measured using an ATP-based assay. (**d**) Representative images of H460-SP spheroids and EthD-1 staining after 6 days of treatment with trimebutine at the indicated concentrations (scale bar = 500 μm). Spheroid size and viability were quantified using GraphPad Prism 8. Data are presented as the mean ± SD of triplicate experiments (*n* = 3). Statistical significance was determined by one-way ANOVA (* *p* < 0.05; ** *p* < 0.01; *** *p* < 0.001; **** *p* < 0.0001).

We compared the effects of trimebutine on parental H460 cells and sphere-derived H460-SP cells using gefitinib and cisplatin as reference compounds. The GI50 values of gefitinib (SI = 1.00) and cisplatin (SI = 1.39) were comparable between H460 and H460-SP cells, whereas trimebutine exhibited markedly greater growth-inhibitory activity in H460-SP cells than in parental H460 cells (SI = 45.55; Figure 1a). While trimebutine had little effect on the proliferation of H460 cells, it significantly reduced the viability of H460-SP cells in a dose-dependent manner (GI50 = 1.099 μM; Figure 1b,c).

Furthermore, to assess selectivity and potential cytotoxicity in non-tumorigenic cells, we evaluated the effects of these compounds in human bronchial epithelial BEAS-2B cells. While cisplatin (GI50 = 5.04 ± 6.23 μM) and gefitinib (GI50 = 13.28 ± 1.96 μM) induced noticeable baseline toxicity, trimebutine showed minimal cytotoxicity in BEAS-2B cells (GI50 > 50 μM; Supplementary Figure S4), indicating selective growth inhibition toward sphere-forming cells over normal epithelial cells.

To further evaluate its effects on lung cancer stem cell-like properties, H460-SP cells were treated with increasing concentrations of trimebutine, and spheroid growth and sphere-forming capacity were assessed on day 6. Trimebutine treatment resulted in a dose-dependent reduction in spheroid size and a concomitant increase in EthD-1 fluorescence intensity, indicating enhanced cell death and reduced viability within the spheroids (Figure 1d). Collectively, these findings demonstrate that H460-SP cells are preferentially sensitive to trimebutine, suggesting its potential efficacy against lung cancer cell populations.

### 2.2. Trimebutine Decreases the Stemness of Lung CSC-like Cells via Modulation of Hippo Signaling Pathway

**Figure 2:**
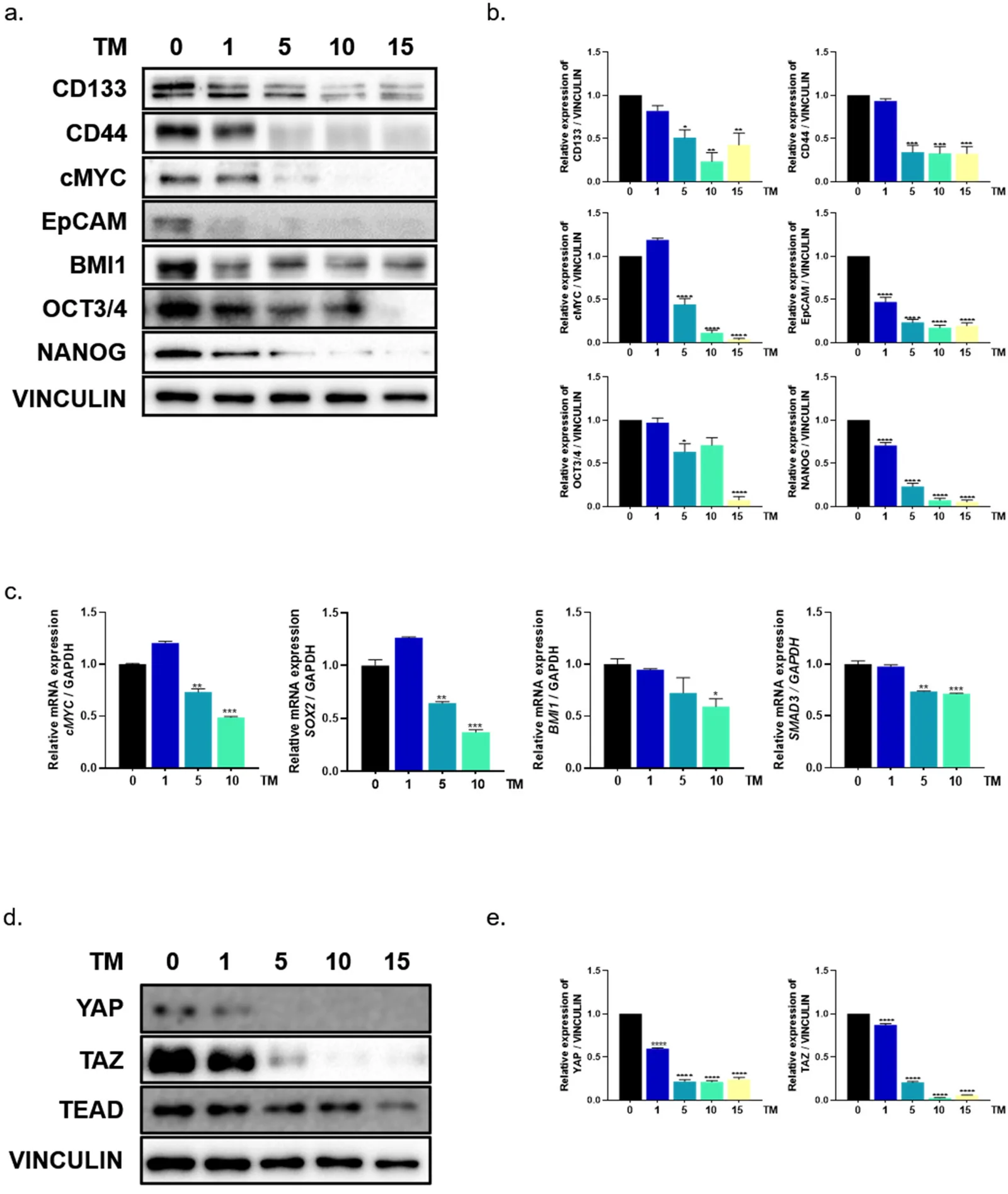
Trimebutine reduces stemness in lung CSC-like cells. (**a**,**b**) Western blot analysis of stemness marker protein levels in H460-SP cells treated with the indicated concentrations of trimebutine for 3 days. (**a**) Representative blots and (**b**) quantitative analysis of protein expression normalized to vinculin. (**c**) Relative mRNA expression levels of transcription factors (cMYC, SOX2, BMI1) and the stemness-associated factor (SMAD3) in trimebutine-treated H460-SP cells, as determined by real-time PCR. Expression levels were normalized to GAPDH. (**d**,**e**) Immunoblots of Hippo signaling pathway proteins in H460-SP cell treated with trimebutine for 3 days. (**d**) Representative blots and (**e**) quantitative analysis are shown. Bar colors represent TM concentrations: black (0 µM, vehicle control), blue (1 µM), cyan (5 µM), light green (10 µM), and yellow (15 µM). Data are presented as the mean ± SD of triplicate experiments (*n* = 3). Statistical significance was determined by one-way ANOVA (* *p* < 0.05; ** *p* < 0.01; *** *p* < 0.001; **** *p* < 0.0001).

To investigate the effect of trimebutine on cancer stemness, H460-SP cells were treated with increasing concentrations of trimebutine. As shown in Figure 2a,b, trimebutine dose-dependently reduced the protein expression of CSC-associated surface markers, including CD133 and CD44, as well as several key stemness-related regulators, such as c-MYC, EpCAM, BMI1, OCT3/4, and NANOG. Consistent with these findings, quantitative RT-PCR analysis revealed that trimebutine significantly decreased the mRNA expression levels of stemness-associated transcription factors, including cMYC, SOX2, and BMI1, together with SMAD3 (Figure 2c).

To further elucidate the underlying mechanism, we examined signaling pathways affected by trimebutine. As shown in Figure 2d,e, trimebutine markedly reduced the protein levels of YAP, TAZ, and TEAD, core components of the Hippo signaling pathway. Collectively, these findings indicate that trimebutine suppresses CSC-associated markers and stemness-related gene expression in lung cancer stem-like sphere cultures, likely through inhibition of Hippo signaling, thereby impairing the maintenance of lung cancer stem-like cell properties.

### 2.3. Trimebutine Decreases the Stemness of Lung CSC-like Cells Through Calcium and Potassium Channels

**Figure 3:**
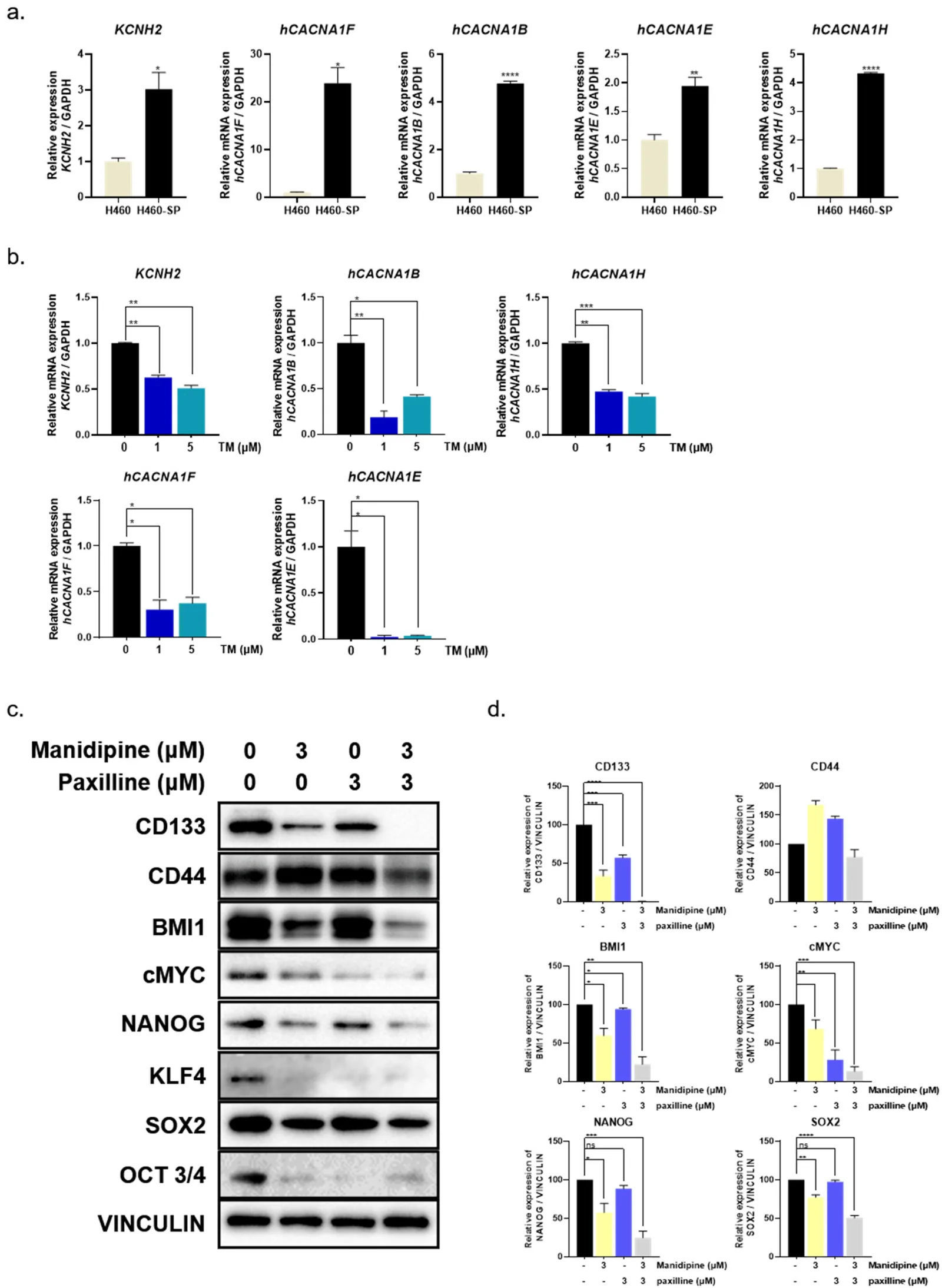
Trimebutine modulates ion channel expression and reduces stemness. (**a**) Relative mRNA expression levels of the potassium channel gene KCNH2 and calcium channel genes (CACNA1F, CACNA1B, CACNA1E, and CACNA1H) in H460 and H460-SP cells. (**b**) Relative mRNA expression levels of KCNH2 and calcium channel genes in H460-SP cells following treatment with trimebutine at the indicated concentrations for 3 days. (**c**) Western blot analysis of stemness-associated protein expression in H460-SP cells treated with a calcium channel blocker (manidipine) and a potassium channel blocker (paxilline), either alone or in combination. (**d**) Quantitative analysis of protein expression. Bar colors indicate experimental conditions: (**a**) beige (H460) and black (H460-SP); (**b**) black (0 µM), blue (1 µM), and cyan (5 µM TM); (**d**) black (vehicle control), yellow (3 µM manidipine), blue (3 µM paxilline), and grey (co-treatment of 3 µM manidipine and 3 µM paxilline). Data are presented as the mean ± SD of triplicate experiments (*n* = 3). Statistical significance was determined by two-tailed Student’s *t*-test or one-way ANOVA (* *p* < 0.05; ** *p* < 0.01; *** *p* < 0.001; **** *p* < 0.0001).

Trimebutine is an opioid receptor–targeting GPCR agonist, and GPCR signaling is known to modulate calcium and potassium channel activity. Therefore, we examined the expression of ion channel–related transcripts in CSC-like cells. As shown in Figure 3a, the relative mRNA levels of KCNH2 and several calcium channel genes (CACNA1F, CACNA1B, CACNA1E, and CACNA1H) were significantly elevated in H460-SP cells compared with parental H460 cells. Treatment with trimebutine dose-dependently reduced the expression of these ion channel genes in H460-SP cells (Figure 3b).

**Figure 4:**
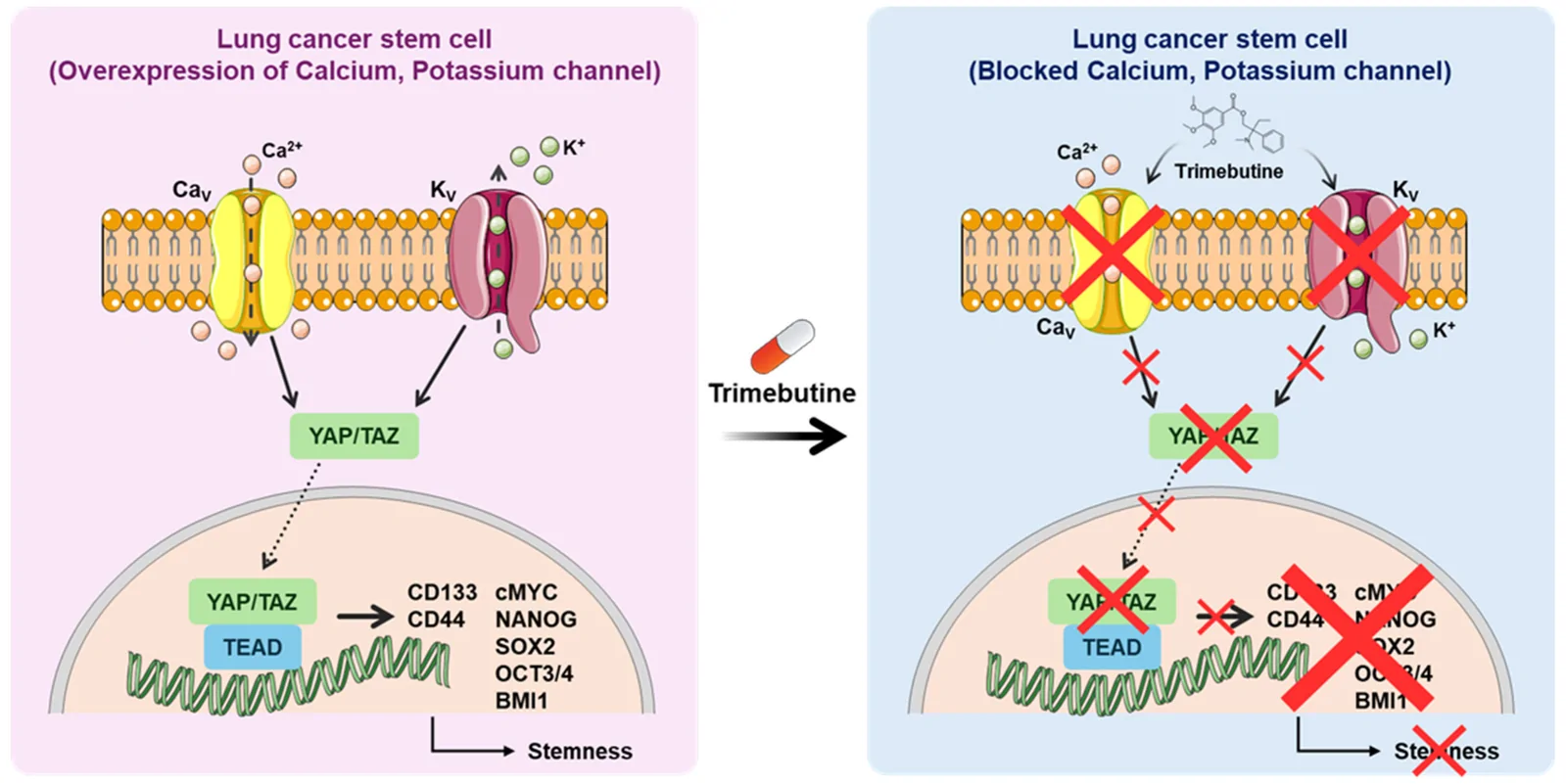
Schematic representation of the proposed mechanism. In the lung CSC microenvironment, elevated activity of calcium and potassium channels promotes the nuclear translocation of YAP/TAZ, enabling their interaction with TEAD to sustain stemness-related transcriptional programs. Trimebutine simultaneously inhibits both ion channels, thereby disrupting upstream signaling inputs required for YAP/TAZ stabilization. This dual inhibition facilitates YAP/TAZ inactivation and degradation, leading to suppression of stemness-associated markers and induction of cell death.

To further investigate whether pharmacological inhibition of these channels functionally contributes to CSC-like properties rather than representing a secondary consequence of non-specific cytotoxicity, parental H460 and sphere-derived H460-SP cells were treated with a panel of various ion channel modulators. Among the tested modulators, the calcium channel blocker manidipine (GI50 = 2.32 μM in H460-SP vs. 8.39 μM in H460) and the BK channel blocker paxilline (GI50 = 6.15 μM in H460-SP vs. 11.52 μM in H460) preferentially reduced the viability of H460-SP cells compared to parental H460 cells (Supplementary Figures S6 and S7), whereas other channel modulators had minimal effects. Furthermore, following 72 h of treatment, pharmacological inhibition of these channels markedly reduced the expression of stemness-associated markers (CD133, CD44, BMI1, c-MYC, NANOG, KLF4, SOX2, and OCT3/4), with the combined treatment producing a greater suppressive effect than either agent alone (Figure 3c,d). In addition, manidipine and paxilline co-treatment suppressed YAP and TAZ protein levels in H460-SP spheres (Supplementary Figure S5) functionally recapitulating the downstream inhibitory effects of trimebutine. Collectively, these findings indicate that calcium and potassium channel activity contributes to the maintenance of stemness, and that trimebutine attenuates stem-like properties in lung cancer cells, at least in part, through modulation of ion channel signaling (Figure 4).

### 2.4. Combination of Trimebutine and Cisplatin Exerts a Synergistic Effect on Lung CSC-like Cells

Our previous findings demonstrated the clinical potential of trimebutine for targeting lung CSC-like cells. We therefore hypothesized that a combinatorial strategy would be more effective than monotherapy in maximizing therapeutic efficacy. To test this, we investigated the effects of trimebutine in combination with cisplatin, a first-line chemotherapeutic agent for lung cancer, with a particular focus on the CSC population.

**Figure 5:**
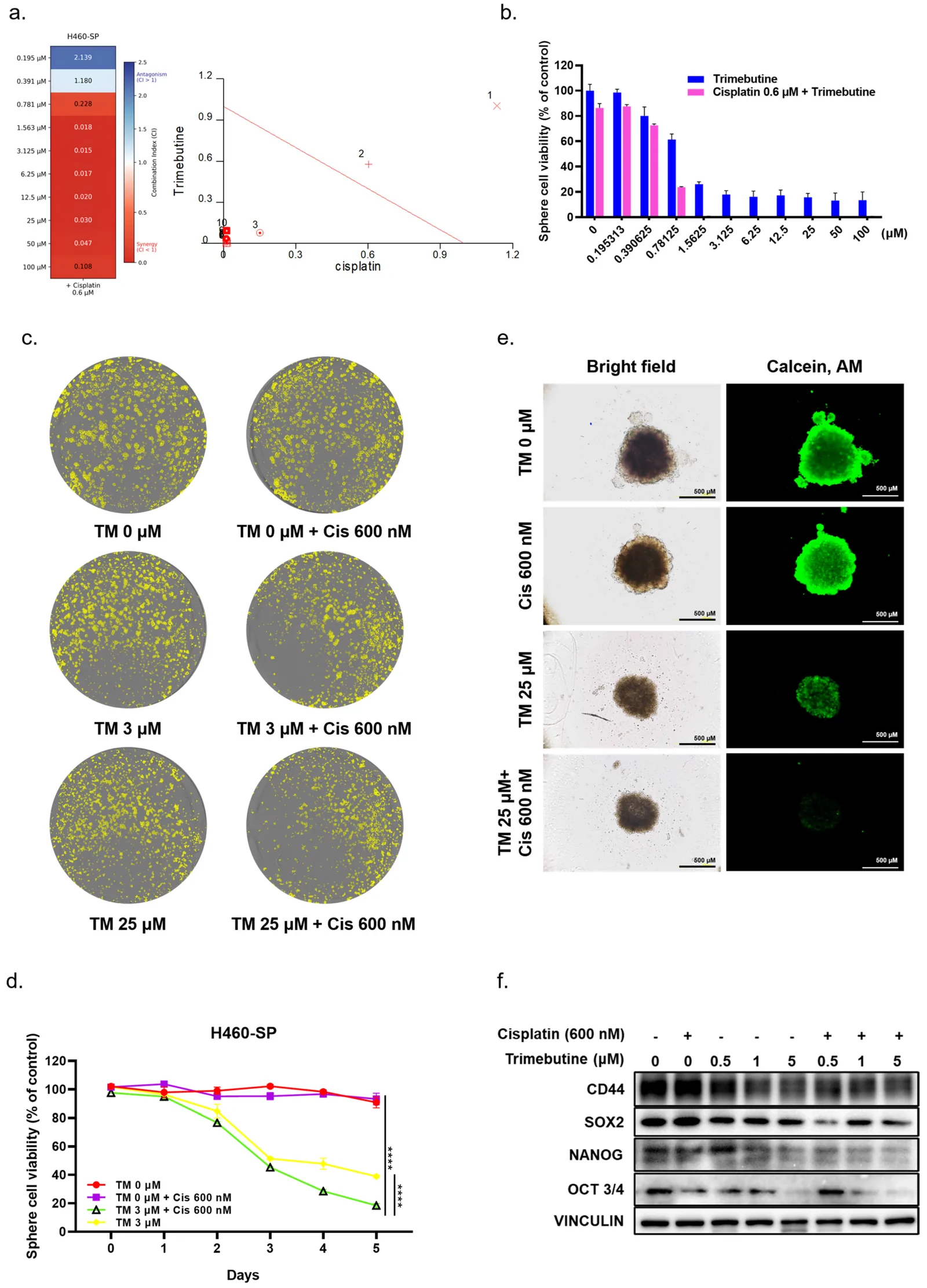
Trimebutine synergizes with cisplatin to inhibit lung CSC-like cells. (**a**) Combination index (CI) heatmap (left) and isobologram analysis (right) of H460-SP spheres treated with combinations of trimebutine and cisplatin, analyzed using the Chou-Talalay method (CI<1 indicates synergy). In the isobologram, the numbers (1–10) indicate combinations of increasing concentration of trimebutine (0.195, 0.391, 0.781, 1.563, 3.125, 6.25, 12.5, 25, 50, and 100 μM) with 0.6 μM cisplatin. (**b**) Cell viability of H460-SP cells treated with trimebutine alone or in combination with cisplatin. (**c**) Representative images showing H460-SP cell proliferation following treatment with trimebutine alone or in combination with cisplatin. (**d**) Quantitative analysis of cell viability following single or combination treatment. (**e**) Representative images of Calcein AM staining in H460-SP cells after 6 days of treatment (scale bar = 500 μm). (**f**) Western blot analysis of stemness-associated protein expression in H460-SP cells treated with trimebutine and cisplatin, alone or in combination. Data are presented as the mean ± SD of triplicate experiments (*n* = 3). Statistical significance was determined by one-way ANOVA or two-way ANOVA (**** *p* < 0.0001).

Given the preferential sensitivity of H460-SP cells to trimebutine (Figure 1a), we next assessed whether combining trimebutine with cisplatin would further enhance the inhibition of CSC-like cells. A cisplatin concentration that reduced H460-SP cell viability to approximately 10–20% (600 nM) was selected, and combination effects were analyzed using CompuSyn based on the Chou–Talalay method. Combination index (CI) and isobologram analyses revealed synergistic interactions between cisplatin (600 nM) and trimebutine, beginning at 700 nM (Figure 5a,b).

Consistent with these findings, co-treatment reduced H460-SP cell viability and proliferation to a greater extent than trimebutine alone and markedly decreased Calcein AM–positive live cells (Figure 5c–e). Based on these results, we further evaluated the effects of combined treatment with trimebutine (0.5, 1, and 5 μM) and cisplatin (600 nM) on stemness-associated protein expression by Western blotting. CD44 expression was particularly sensitive to the combination, showing a pronounced decrease even at the lowest tested concentrations. In addition, the expression levels of key stemness-related proteins, including SOX2, NANOG, OCT3/4, and KLF4, were downregulated in a dose-dependent manner, consistent with the observed synergistic interaction between the two agents.

### 2.5. Combined Treatment with Trimebutine and Cisplatin Suppresses Survival and Stemness in Patient-Derived Lung Tumor Organoids

To validate these findings in a clinically relevant setting, patient-derived lung tumor organoids (PDOs; SNU-2627-CO, derived from non-small cell lung cancer, Supplementary Table S2) were treated with trimebutine and cisplatin, either alone or in combination. Given the intrinsic heterogeneity of PDOs, trimebutine was administered at 5 μM to ensure consistent efficacy targeting lung CSC-like subpopulations across diverse cellular. For combination studies, a cisplatin concentration of 1 μM was selected based on prior dose–response analyses to assess potential synergistic effects in a clinically relevant context.

**Figure 6:**
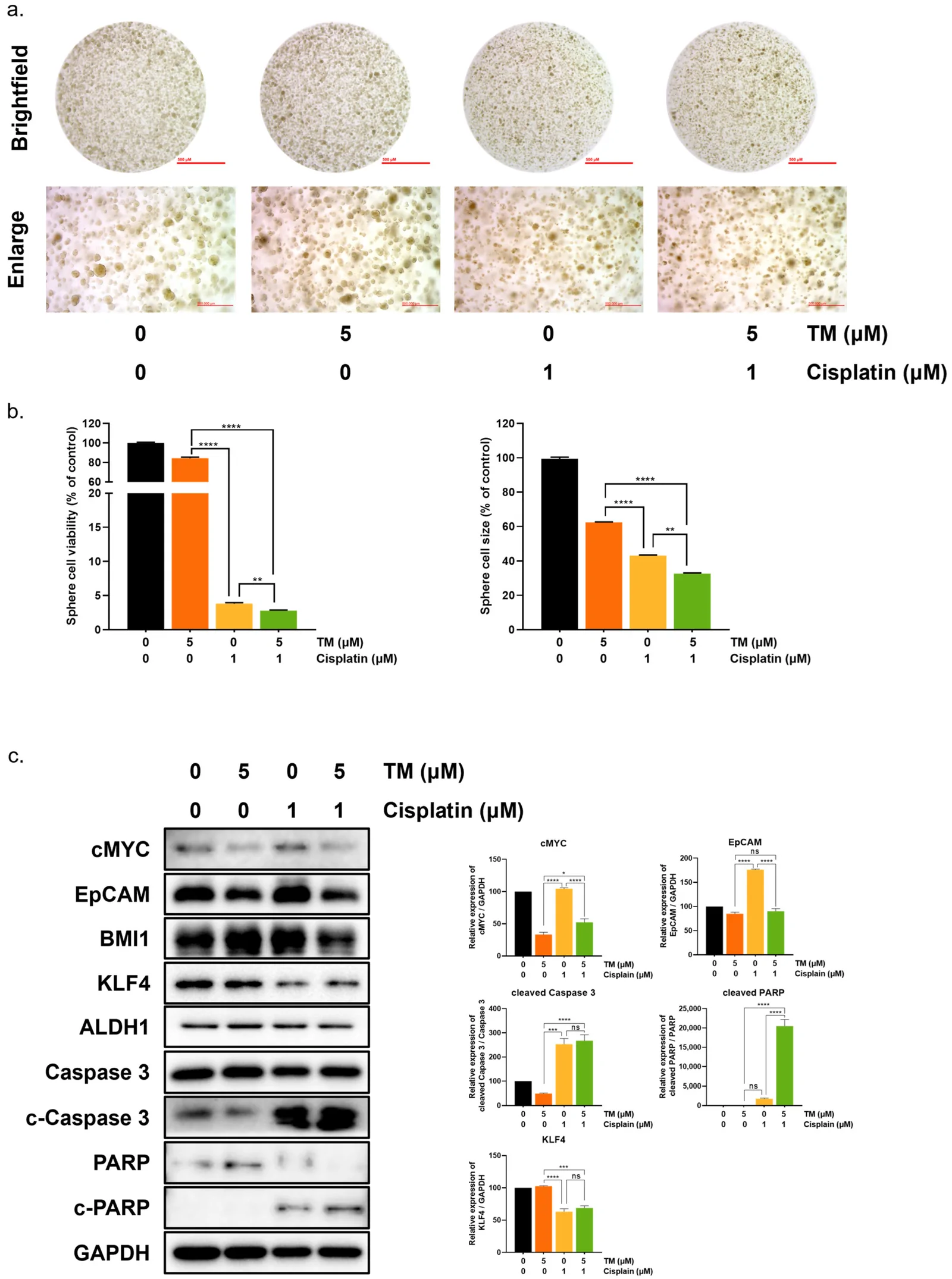
Combination treatment with trimebutine and cisplatin suppresses stemness in patient-derived tumor organoids. (**a**,**b**) PDOs were treated with trimebutine and cisplatin, either alone or in combination, for 72 h. (**a**) Representative brightfield and enlarged images of PDOs (scale bar = 500 μm for both panels). (**b**) Quantification of organoid size and cell viability. (**c**) Western blot analysis of stemness- and apoptosis-related proteins in PDOs treated with trimebutine and/or cisplatin for 72 h. Representative blots (left) and quantitative analysis (right) are shown. The bar colors represent treatment groups: black (control; 0 µM TM, 0 µM Cisplatin), orange (5 µM TM), yellow (1 µM Cisplatin), and green (combination of 5 µM TM and 1 µM Cisplatin). Data are presented as the mean ± SEM of three independent experiments. Statistical significance was determined by one-way ANOVA (* *p* < 0.05; ** *p* < 0.01; *** *p* < 0.001; **** *p* < 0.0001).

Consistent with our expectations, trimebutine alone exerted a modest effect on overall cell viability, whereas cisplatin treatment induced substantial cytotoxicity. Notably, co-treatment further potentiated this effect, resulting in the lowest cell viability among all groups. Quantitative analysis of organoid size demonstrated that the combination therapy more effectively suppressed organoid growth than either monotherapy, with the greatest reduction observed in the co-treatment group (Figure 6a,b).

At the molecular level, combination treatment led to a pronounced downregulation of stemness-associated proteins (c-MYC, EpCAM, BMI1, KLF4, and ALDH1) along with a marked induction of apoptotic markers (cleaved caspase-3 and cleaved PARP) in SNU-2627-CO organoids (Figure 6c). Furthermore, in the independent adenocarcinoma organoid model (SNU-2693-CO), co-treatment with trimebutine robustly abrogated cisplatin-induced upregulation of c-MYC and suppressed key stemness regulators, including KLF4 and EpCAM (Supplementary Figure S8). These results indicate that the combination of trimebutine and cisplatin consistently reduces PDO viability and growth across distinct patient-derived models by counteracting cisplatin-induced stemness enrichment and promoting cell death.

Collectively, these findings highlight the clinical potential of trimebutine as a novel therapeutic agent targeting lung CSC-like cells and support its use in combination strategies aimed at reducing tumor recurrence.

## 3. Discussion

Therapeutic resistance and recurrence remain major challenges in lung cancer, and accumulating evidence implicates stem cell–like subpopulations in relapse following cytotoxic or targeted therapy. In this study, we evaluated a focused panel of 21 candidate compounds prioritized from a primary high-throughput screen of 1018 FDA-approved drugs in an ovarian CSC model [14] to investigate their therapeutic relevance in lung cancer stem-like models. Notably, when tested in 3D H460-SP sphere cultures, only 4 of the 21 candidates exhibited potent and selective growth-inhibitory activity against lung cancer stem-like cells. These results suggest that stemness-associated signaling pathways differ between ovarian and lung cancers, resulting in cancer-type-specific drug responses. Among these validated hits, trimebutine, an enkephalin analogue that modulates gastrointestinal motility via opioid receptors, signals through G protein–coupled receptor (GPCR) pathways [15], which are frequently dysregulated in cancer and converge on key regulators of proliferation, survival, and stemness [16,17]. Consistent with this, trimebutine displayed preferential activity under CSC-enriched conditions and enhanced the anti–stemness effects of cisplatin; their combination produced greater inhibition of lung CSC-like cells than either agent alone, supporting further evaluation of trimebutine-based combinations to mitigate CSC-like subpopulation-driven resistance and recurrence.

Trimebutine is also known to regulate ion channels [18,19], and our data show that it preferentially attenuates stemness associated features in lung CSC like cells while increasing cell death in sphere cultures. Mechanistically, trimebutine acts as a weak non-selective agonist for peripheral opioid GPCRs (μ-, δ-, and κ-opioid receptors), which are known to modulate intracellular ion fluxes via G_βγ_ and G_αi_ subunit-mediated regulation of voltage-gated calcium channels (VGCCs) and potassium channels. In line with this cascade, our pharmacological perturbation experiments demonstrated that selective blockade of calcium (manidipine) and BK potassium (paxilline) channels directly downregulates YAP and TAZ protein levels (Supplementary Figure S5b), recapitulating the inhibitory effect of trimebutine on CSC properties. Given that multiple ion channels are overexpressed in CSC-like populations [20,21], and that the coordinated activity of calcium and potassium channels governs intracellular signaling and the physical microenvironment, these channels are likely to play a critical role in regulating the Hippo signaling pathway, which is essential for the maintenance of lung CSC-like populations [22,23]. These findings support a model in which trimebutine modulates CSC-like cell maintenance by targeting a network of ion channel pathways [18]. In our CSC-like model, the combination of trimebutine and cisplatin exhibited marked synergistic effects. While low-dose trimebutine monotherapy had a minimal impact on lung CSC-like cells viability, co-treatment with cisplatin dramatically reduced cell survival and further suppressed proliferation. Protein level changes, however, did not scale linearly with cytotoxicity. Although cell viability dropped sharply at higher trimebutine concentrations, the most pronounced synergistic reduction in stemness marker expression occurred at lower dose, reaching a plateau as concentrations increased.

This non monotonic, bell-shaped concentration–response likely reflects both physicochemical and biological mechanisms. At lower concentrations, trimebutine likey remains predominantly monomeric and membrane-permeable, efficiently suppressing cisplatin-induced stemness programs, whereas at higher concentrations it may exceed its critical aggregation threshold [24], forming colloidal aggregates that limit effective intracellular exposure. In parallel, higher doses in combination with cisplatin may engage compensatory survival pathways and paradoxical feedback loops [25] commonly observed in oncologic co treatments, blunting further suppression of stemness markers despite increased cytotoxicity. Together, these observations point to an optimal therapeutic window at lower doses, where trimebutine most effectively neutralizes resistance pathways while avoiding aggregation driven and adaptive constraints.

In contrast to trimebutine monotherapy, which systematically downregulates core stemness factors (as described in Results), cisplatin treatment alone substantially reduced overall cell viability but simultaneously enriched chemoresistant CSC-like cells, as evidenced by upregulation of CD44, NANOG, and SOX2 alongside downregulation of OCT3/4 in the surviving fraction, consistent with prior reports of cisplatin induced CSC reprogramming [26]. Conversely, trimebutine co-treatment or monotherapy dose-dependently counteracted this phenomenon, achieving robust inhibition of CD44, SOX2, NANOG, and OCT3/4. Notably, at this concentration the combination did not confer an additive reduction in marker expression, but it did abrogate cisplatin induced CSC enrichment, indicating that trimebutine primarily targets core stemness transcriptional programs while cisplatin provides bulk cytotoxicity. In patient-derived lung tumor organoids, therapeutic responses varied predictably according to histological subtypes (Supplementary Table S2). In the NSCLC-derived SNU-2627-CO model, combination therapy broadly suppressed multiple stemness regulators (c-MYC, EpCAM, BMI1, KLF4, and ALDH1) and markedly induced apoptotic cleavage (Figure 5) [27,28,29,30]. Similarly, in the independent adenocarcinoma-derived organoid model (SNU-2693-CO; Supplementary Figure S8), cisplatin monotherapy provoked a prominent compensatory upregulation of the key stemness driver c-MYC (152% of control), consistent with prior reports of chemotherapy-induced CSC reprogramming [26,31,32]. Strikingly, co-treatment with trimebutine robustly abrogated this c-MYC induction alongside the downregulation of self-renewal factors including KLF4 and EpCAM [27,28,29,30] and a significant reduction in organoid growth. Together, these findings demonstrate that trimebutine consistently counteracts cisplatin-induced stemness enrichment and reinforces anti-tumor efficacy across diverse patient-derived lung cancer backgrounds.

Several limitations should be acknowledged. First, our CSC like population was operationally defined using sphere derived cultures and stemness marker profiles; additional functional assays, such as serial sphere formation, limiting dilution transplantation, and in vivo tumor initiating capacity, are needed to rigorously validate CSC properties. Second, although our pharmacological perturbation data (Supplementary Figures S5–S7) provide clear functional evidence that calcium and potassium channel blockade directly leads to YAP/TAZ suppression, the precise contribution of individual opioid receptor subtypes, μ, δ, or κ) and their direct biophysical coupling to specific channel subunits warrant further electrophysiological and genetic dissection. Third, the generalizability of our findings should be tested across a broader panel of lung cancer models, including genetically and histologically diverse subtypes and additional combination regimens.

In summary, our results support trimebutine as a repurposing candidate with preferential activity against lung CSC-like cells and demonstrate that co treatment with cisplatin enhances suppression of CSC-like markers and growth properties. Given trimebutine’s FDA approved status, favorable safety profile, and low systemic toxicity, its integration into standard cisplatin-based regimens could be rapidly advanced into early phase clinical testing. Such a strategy offers a practical route to selectively target resilient CSC-like subpopulations, with the potential to reduce relapse and overcome chemoresistance without substantially increasing treatment related toxicity. From a health system perspective, this repurposing approach may also provide a cost effective alternative to de novo drug development, offering a scalable means to alleviate the growing economic burden of lung cancer care.

## 4. Materials and Methods

### 4.1. Cell Culture

All experimental procedures involving cell lines were performed in accordance with standard ethical guidelines for biomedical research. The human lung cancer cell line H460 and patients-derived lung cancer organoid (SNU-2693-CO and SNU-2627-CO) were purchased from KCLB (Seoul, Republic of Korea). The detailed clinicopathological characteristics of the patient donors are summarized in Supplementary Table S2. Because only commercially available, established human cell lines (H460) were used, this study was exempt from institutional review board (IRB) review according to institutional policy. H460 cell lines were maintained in RPMI-1640 medium (Hyclone, Logan, UT, USA) supplemented with 10% fetal bovine serum (Hyclone) and 1% penicillin/streptomycin (Hyclone). The cells were cultured in 100 mm^2^ plates (Corning, NY, USA), and the medium was changed every 2–3 days. H460-SP cells were cultured in DMEM/F12 10 mL (Gibco, Gaithersburg, MD, USA), supplemented with 2% B27 serum-free supplement, 1% penicillin/streptomycin (Hyclone), 10 ng/mL basic fibroblast growth factor (R&D Systems, Minneapolis, MN, USA), and human epidermal growth factor (R&D Systems, Minneapolis, MN, USA) in Ultra-Low-Attachment 100 mm^2^ plates (Corning). The medium was changed every 2–3 days. Sphere-forming cells were dissociated into single cells using Accutase (Gibco, Gaithersburg, MD, USA). SNU-2693-CO organoid cells were cultured in extracellular matrix (ECM) domes overlaid with organoid culture medium consisting of 40% basal medium, 50% L-WRN conditioned medium, 1× B27 supplement, 5 ng/mL human epidermal growth factor (R&D Systems, Minneapolis, MN, USA), 10 mM nicotinamide, 1.25 mM N-acetylcysteine, and 500 nM A83-01. The medium was changed every 2–3 days. Trimebutine maleate, cisplatin, manidipine hydrochloride, paxilline, tetrodotoxin (TTX), 4-aminopyridine (4-AP), glibenclamide, minoxidil, and cromakalim were purchased from Sigma-Aldrich (St. Louis, MO, USA) or Selleck Chemicals (Houston, TX, USA) and dissolved in DMSO or sterile water according to the manufacturer’s instructions.

### 4.2. Secondary Drug Screening and Hit Validation

A focused panel of 21 candidate compounds, prioritized from a primary high-throughput screen of 1018 FDA-approved drugs (Selleck Chemicals, Houston, TX, USA) based on sphere-disrupting activity [14], was evaluated in the 3D H460-SP lung cancer stem-like sphere model (Supplementary Table S1). Dissociated single H460-SP cells were seeded into 96-well ultra-low attachment (ULA) round-bottom plates (Corning, Corning, NY, USA) at a density of 3000 cells/well in stem cell culture medium. After 24 h of sphere initiation, candidate compounds were added at concentrations of 1 and 5 μM (final DMSO concentration 0.5%). Vehicle (0.5% DMSO) and standard cytotoxic agents (gefitinib) were used as negative and positive controls, respectively. The primary screen was performed in biological triplicates (*n* = 3) to ensure assay reproducibility. After 6 days of treatment, sphere cell viability was determined using the CellTiter-Glo 3D Cell Viability Assay (Promega) on a Synergy H1 plate reader (BioTek). Hits were defined as compounds demonstrating > 50% viability inhibition in H460-SP spheres alongside a high Selectivity Index (SI > 10) over parental H460 cells.

### 4.3. Cell Viability Assay

H460-SP cells were cultured in Corning Ultra-Low Attachment round-bottom 96-well plates at a density of 3000 viable cells per well, followed by centrifugation at 2000 rpm for 5 min. H460 cells were seeded in standard 96-well cell culture plates (Corning) at a density of 3000 cells per well. After 24 h, trimebutine and cisplatin were added to each well. The medium was changed after 72 h for H460-SP cells. The viability of sphere-forming cells was evaluated after 7 days using the CellTiter-Glo assay (Promega, Madison, WI, USA), and luciferase activity was measured using a Synergy H1 plate reader (Biotek, Winooski, VT, USA). The viability of H460 cells was assessed using the CellTiter-Glo assay (Promega) 3 days after treatment with compounds.

### 4.4. Sphere Size Analysis

H460-SP cells were cultured in ultra-low-attachment round-bottom 96-well plates (Corning) at a density of 3000 viable cells per well. The samples were centrifuged at 2000 rpm for 5 min and incubated at 37 °C. After 24 h, the test compounds were added. After 3 days, the medium was replaced with a fresh medium containing the same compound concentration. After 6 days, the sphere-forming cells were imaged using a Cytation 5 system (Biotek). Sphere sizes were quantified using the ImageJ software (version 1.53; National Institutes of Health, Bethesda, MD, USA).

### 4.5. Western Blot Analysis

H460-SP and A549-SP cells were cultured in Corning Ultra-Low Attachment six-well plates at a density of 5 × 10^5^ viable cells/well in an appropriate growth medium. After 24 h, the cells were treated with the compounds, incubated for 72 h, and harvested. Whole-cell lysates were obtained using a protein extraction solution, specifically radioimmunoprecipitation assay buffer, supplemented with phosphatase and protease inhibitor cocktail. The resulting cell lysates were separated via sodium dodecyl sulfate–polyacrylamide gel electrophoresis and transferred onto polyvinylidene fluoride membranes for Western blot analysis. After blocking with 5% bovine serum albumin (BSA), the membranes were incubated overnight at 4 °C with primary antibodies diluted in 2.5% BSA, followed by incubation for 1 h at 25 °C with the corresponding horseradish peroxidase (HRP)-conjugated secondary antibodies. The primary antibodies used in this study included CD133 (Abcam, Cambridge, MA, USA, ab19898), CD44 (Cell Signaling Technology, Danvers, MA, USA, #5640S), KLF4 (GeneTex, San Antonio, TX, USA, GTX101508), CMYC (Cell Signaling Technology, #5605S), BMI1 (Cell Signaling Technology, #6964S), OCT 3/4 (Santa Cruz Biotechnology, Santa Cruz, CA, USA, sc-8682), NANOG (Cell Signaling Technology, Danvers, MA, USA, #3580S), VINCULIN (Santa Cruz Biotechnology, sc-73614).

The secondary antibodies used were goat anti-mouse IgG-HRP and goat anti-rabbit IgG-HRP, both of which were purchased from Bioss (Woburn, MA, USA). Signals were detected using the SuperSignal™ West Femto Maximum Sensitivity Substrate (Thermo Scientific™, Waltham, MA, USA) and analyzed using LuminoGraph III Lite (ATTO, Tokyo, Japan).

### 4.6. Quantitative RT-PCR

Total RNA was extracted from 1 × 10^6^ using the TRIzol RNA extraction kit (Invitrogen, Carlsbad, CA, USA) according to the manufacturer’s guidelines. Subsequently, 2 μg of RNA was reverse transcribed to synthesize complementary DNA (cDNA) using the High-Capacity cDNA Reverse Transcription Kit (Applied Biosystems, Waltham, MA, USA). The resulting cDNA was amplified using quantitative real-time PCR with PowerUp SYBR Green Master Mix (Applied Biosystems) and a Bio-Rad S1000 Thermal Cycler (Bio-Rad Laboratories, Hercules, CA, USA), using the specified primers included hCACNA1F (F: 5′–AAGGCATTGAGGGCGTTTC–3′, R: 5′–GCATTCGTCCAAGGAACAGC–3′), hCACNA1B (F: 5′–CCCGATGAAATAGGGCTCCG–3′, R: 5′–GACAACGTCCGCAAATAC–3′), hCACNA1H (F: 5′–GCGAGGAGAACCCGTTCATC–3′, R: 5′–GAGCGGCACACGTTGTAGT–3′), hCACNA1E (F: 5′–CAATACTCACGTGGACCTGAGGACC–3′, R: 5′–GAAGAGCAGAAGGCCAATCTGCAG–3′), hKCNH2 (F: 5′–TTCGACCTGCTCATCTTCGG–3′, R: 5′–CGATGCGTGAGTCCATGTGT–3′), GAPDH (F: 5′–GGAGCCAAAAGGGTCATCAT–3′, R: 5′–GTGATGGCATGGACTGTGGT–3′). GAPDH was used as the reference gene for normalization. The results were expressed relative to those of the control group using the ΔΔCt method.

### 4.7. Combination Index (CI) Assay

CI was calculated using CompuSyn software (version 1.0; COMBOSYN, Paramus, NJ, USA) following the Chou–Talalay method. The median effective dose (ED50) was determined by obtaining the slope and intercept values from the dose–response curve for each drug. Based on these ED50 values, the CI value, a key indicator of the combination effects, was evaluated. Finally, isobologram analysis and CI fraction-affected (Fa) plots were used to determine whether the interaction between the two drugs was synergistic, additive, or antagonistic. These two methods yielded consistent results for the drug interactions. A CI value less than 1 indicates a synergistic effect, equal to 1 indicates an additive effect, and greater than 1 indicates an antagonistic effect.

### 4.8. Sphere Proliferation Assay

H460-SP cells were seeded into Corning Ultra-Low Attachment flat-bottom 96-well plates at a density of 5000 viable cells per well and cultured in CSC medium. After 24 h, the H460-SP cells were treated with the indicated compounds. The spheres were cultured for 60 h. After 60 h, sphere formation was analyzed using an IncuCyte (BioTek).

### 4.9. Live/Dead Fluorescence Staining of the Spheres

Fluorescence staining was performed using a LIVE/DEAD assay kit (Invitrogen) to visualize cell distribution and viability. H460-SP cells were seeded into Corning Ultra-Low Attachment round-bottom 96-well plates at a density of 3000 viable cells and cultured in CSC medium. After 24 h, the H460-SP cells were treated with the compound. After 72 h, the medium was replaced with fresh medium containing the same concentration of the compounds. After 6 days, the cells were incubated with 2 μM Calcein AM and 4 μM EthD-1 in phosphate-buffered saline for 30 min. Fluorescence signals were captured using a fluorescence microscope (Nikon ECLIPSE Ti2 (Nikon Corporation, Tokyo, Japan)). Live cells emitted green fluorescence, whereas the dead cells emitted red fluorescence.

### 4.10. Determination of Selectivity Index (SI)

The 50% growth inhibition (${\mathrm{GI}}_{50}$) values for each compound against parental H460 cells and H460-SP sphere cells were determined from concentration–response curves via nonlinear regression analysis using GraphPad Prism 8 (GraphPad Software, San Diego, CA, USA). To quantify the preferential anti-stemness efficacy of the compounds, the Selectivity Index (SI) was calculated using the following equation:

$$\mathrm{Selectivity}\;\mathrm{Index}\;(\mathrm{SI})=\frac{{\mathrm{GI}}_{50}\;(\mathrm{Parental}\;H460)}{{\mathrm{GI}}_{50}\;(\mathrm{H460-SP}\;\mathrm{Sphere})}$$

An SI>1.0 indicates preferential selectivity toward lung cancer stem-like cells (CSC-like cells) over non-stem parental tumor cells.

### 4.11. Statistical Analysis

Data are reported as mean ± standard deviation (SD) derived from three or more independent experiments. Statistical significance was assessed using the *t*-test with GraphPad Prism 8 (GraphPad Software, San Diego, CA, USA). Statistical significance was set at *p* < 0.05.

## 5. Patents

A Korean patent application was filed based on this manuscript (10-2026-0003428).

## Data Availability

The original contributions presented in this study are included in the article/Supplementary Materials. Further inquiries can be directed to the corresponding authors.

## Supplementary Materials

The following supporting information can be downloaded at: https://www.mdpi.com/article/10.3390/ijms27188211/s1.

## Abbreviations

The following abbreviations are used in this manuscript:

3D	Three-Dimensional
ALDH1	Aldehyde Dehydrogenase 1
BMI1	B cell-specific Moloney murine leukemia virus integration site 1
CACNA	Calcium Voltage-Gated Channel Subunit Alpha
CD133	Cluster of Differentiation 133
CD44	Cluster of Differentiation 44
CI	Combination Index
c-MYC	MYC Proto-Oncogene, BHLH Transcription Factor
CSC	Cancer Stem Cell
EGF	Epidermal Growth Factor
EpCAM	Epithelial Cell Adhesion Molecule
EthD-1	Ethidium Homodimer-1
FDA	Food and Drug Administration
GI50	50% Growth Inhibitory Concentration
GPCR	G Protein-Coupled Receptor
KCNH2	Potassium Voltage-Gated Channel Subfamily H Member 2
KLF4	Kruppel-Like Factor 4
mRNA	Messenger Ribonucleic Acid
NANOG	Nanog Homeobox
NSCLC	Non-Small Cell Lung Cancer
OCT3/4	POU Class 5 Homeobox 1
PARP	Poly (ADP-Ribose) Polymerase
PDO	Patient-Derived Organoid
RT-PCR	Reverse Transcription-Polymerase Chain Reaction
SCLC	Small Cell Lung Cancer
SMAD3	SMAD Family Member 3
SOX2	SRY-Box Transcription Factor 2
TAZ	Transcriptional Coactivator with PDZ-Binding Motif
TEAD	TEA Domain Transcription Factor
YAP	Yes-Associated Protein
