# Supplementary material for: Targeting Lung Cancer Stem Cells with Trimebutine: Synergistic Antitumor Activity in Combination with Cisplatin
Source: Int J Mol Sci. 2026 Sep 15;27(18):8211. doi: 10.3390/ijms27188211 (PMC13607826; doi:10.3390/ijms27188211)
Supplement: Supplementary file 1 [file ijms-27-08211-s001.zip › ijms-4516569-supplementary.pdf]

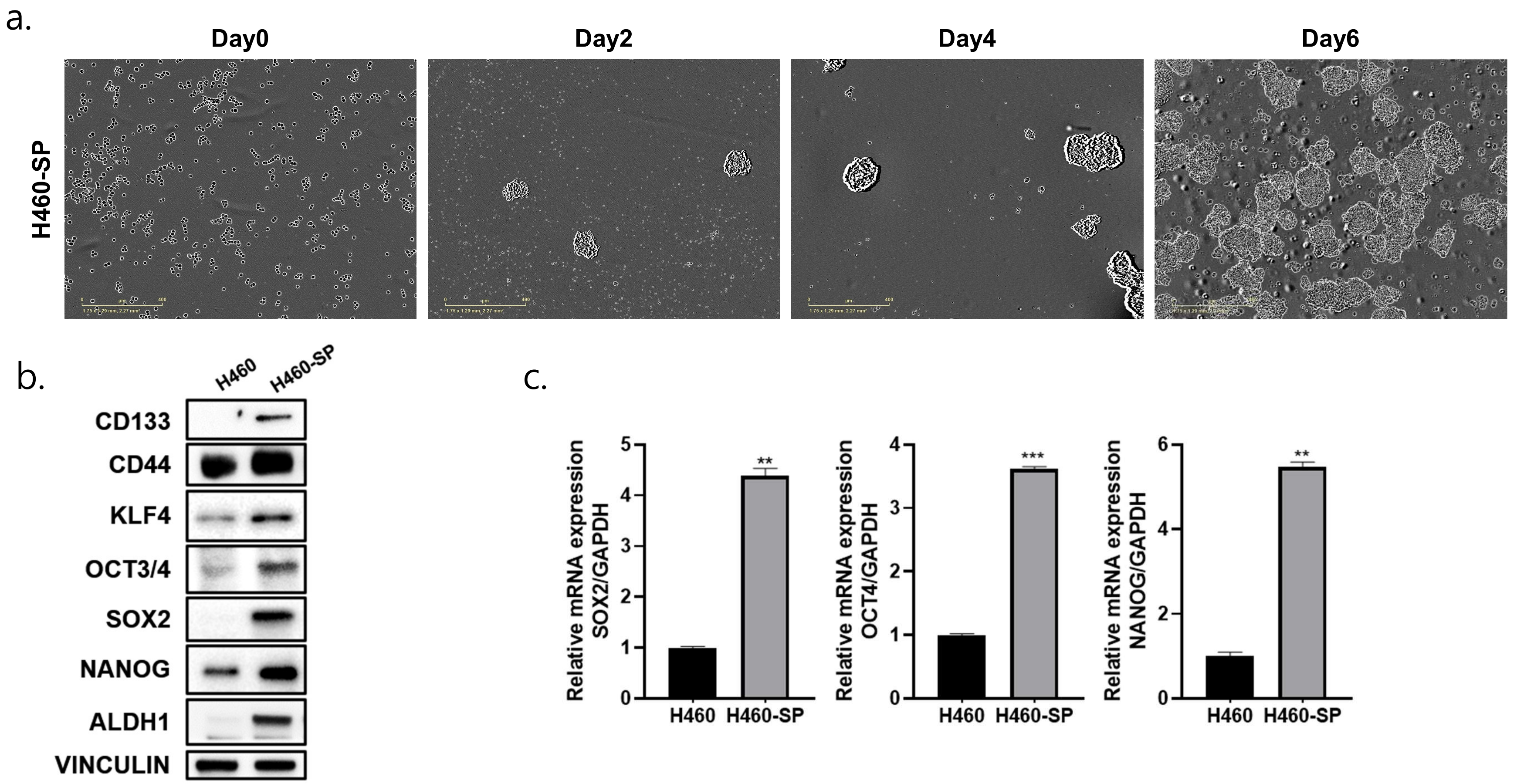

Figure S1. Characterization and validation of stemness properties in H460-SP lung cancer stem-like cells. (a) Representative brightfield images showing the sequential morphological formation and growth of 3D H460-SP spheres at Days 0, 2, 4, and 6 (scale bar = 400 μm).(b) Western blot analysis of stemness marker protein expression (CD133, CD44, KLF4, OCT3/4, SOX2, NANOG, and ALDH1) in parental H460 cells and H460-SP spheres. VINCULIN was used as an internal loading control.(c) Relative mRNA expression levels of core stemness transcription factors (SOX2, OCT4, and NANOG) in parental H460 and H460-SP cells, measured by real-time RT-qPCR and normalized to GAPDH. Data are presented as the mean ± SD of three independent experiments (n = 3). Statistical significance was determined by two-tailed Student's t-test (*p < 0.01, ***p < 0.001 vs. parental H460 cells).

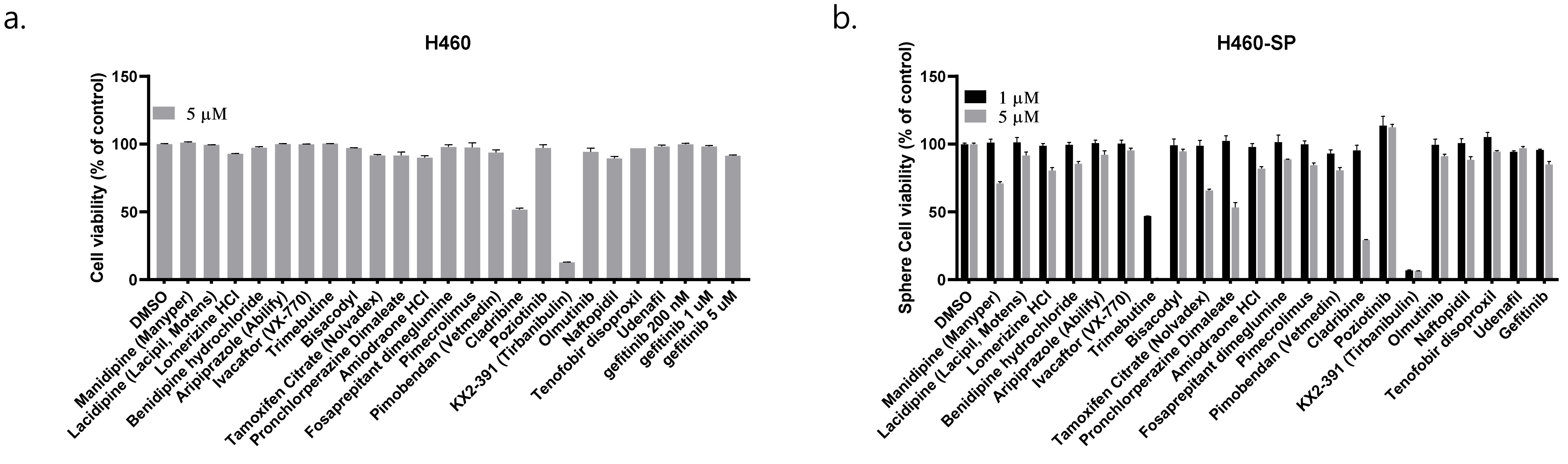

Figure S2. Secondary viability screening and CSC-selective cytotoxicity profile of 21 candidate compounds. (a) Cell viability of parental 2D H460 bulk non-small cell lung cancer cells treated with 21 screened candidate compounds at 5 μM for 72 h, measured by an ATP-based CellTiter-Glo assay. Gefitinib (200 nM, 1 μM, and 5 μM) was used as a comparative control.(b) Relative sphere viability of 3D H460-SP stem-like spheres treated with the 21 candidate compounds at 1 and 5 μM for 6 days. Trimebutine markedly suppressed sphere viability in a concentration-dependent manner while showing minimal cytotoxicity against parental 2D cells, demonstrating preferential selectivity toward CSC-like populations. Data are presented as the mean ± SD of three independent experiments (n = 3). Statistical significance was determined by one-way ANOVA followed by Dunnett's post hoc test (*p < 0.05, **p < 0.01, ***p < 0.001, ****p < 0.0001 vs. vehicle-treated DMSO control).

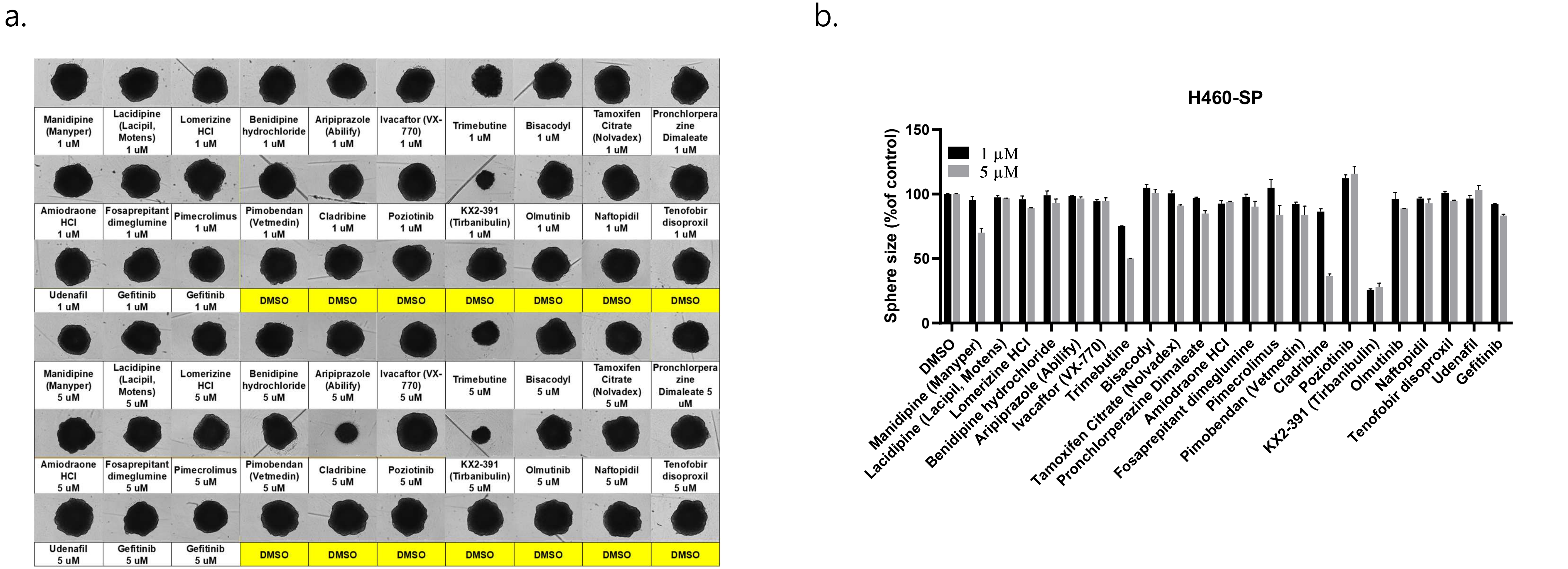

Figure S3. Morphological disruption and size reduction of 3D H460-SP spheres by candidate compounds.(a) Representative brightfield images of 3D H460-SP stem-like spheres after 6 days of treatment with 21 candidate compounds at 1 and 5 μM or vehicle control (DMSO). Gefitinib was used as a comparative control.(b) Quantitative analysis of 3D H460-SP sphere size (diameter/area) following 6 days of treatment with the indicated compounds at 1 and 5 μM, expressed as a percentage relative to the DMSO-treated control. Trimebutine markedly disrupted intact sphere architecture and reduced spheroid size in a dose-dependent manner. Data are presented as the mean ± SD of three independent experiments (n = 3). Statistical significance was determined by one-way ANOVA followed by Dunnett's post hoc test (*$p < 0.05$,**$p < 0.01$, ***$p < 0.001$, ****$p < 0.0001$ vs. vehicle-treated DMSO control).

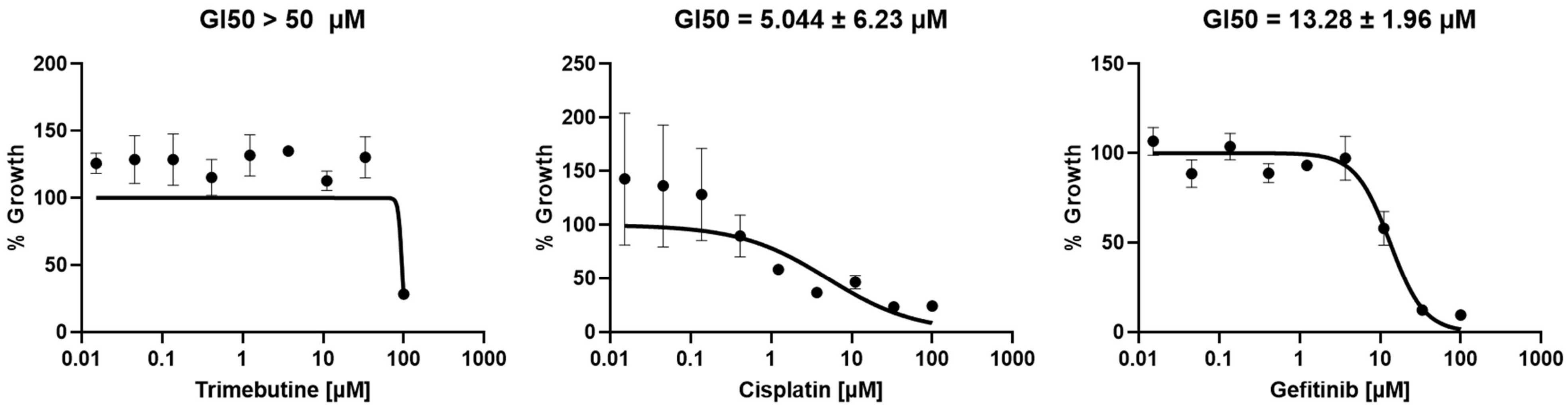

Figure S4. Cytotoxicity evaluation of trimebutine, cisplatin, and gefitinib in normal human bronchial epithelial cells. Dose-response cell viability curves and calculated GI50 values of normal human bronchial epithelial cells (BEAS-2B) treated with trimebutine, cisplatin, or gefitinib for 72 h, measured using an ATP-based CellTiter-Glo assay. Trimebutine exhibited minimal cytotoxicity against normal epithelial cells GI50 > 50 μM, whereas cisplatin and gefitinib displayed cytotoxicity with GI50 values of 5.044 ± 6.23 μM and 13.28 ± 1.96 μM, respectively. GI50 values were determined by non-linear regression analysis using GraphPad Prism 8. Data are presented as the mean ± SD of two independent experiments (n = 2).

Number	Compound
1	Manidipine (Manyper)
2	Lacidipine (Lacipil, Motens)
3	Lomerizine HCl
4	Benidipine hydrochloride
5	Aripiprazole (Abilify)
6	Ivacaftor (VX-770)
7	Trimebutine
8	Bisacodyl
9	Tamoxifen Citrate (Nolvadex)
10	Prochlorperazine Dimaleate
11	Amiodarone HCl
12	Fosaprepitant dimeglumine
13	Pimecrolimus
14	Pimobendan (Vetmedin)
15	Cladribine
16	Poziotinib
17	KX2-391 (Tirbanibulin)
18	Olmutinib
19	Naftopidil
20	Tenofobir disoproxil
21	Udenafil

Table S1. List of 21 prioritized FDA-approved drug candidates evaluated for anti-stemness efficacy in 3D H460-SP sphere models.

a.

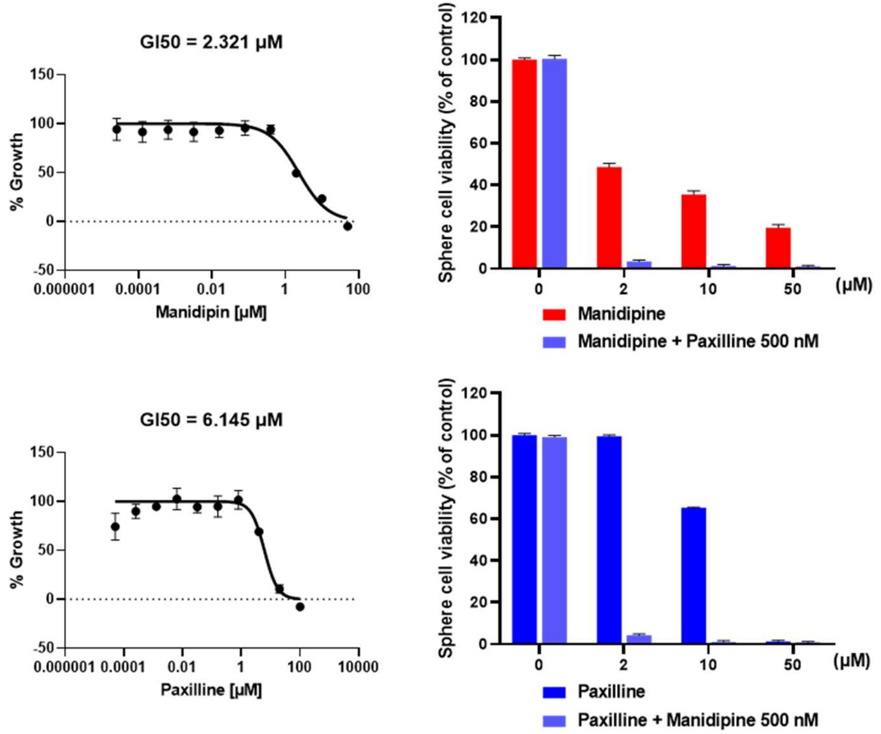

b.

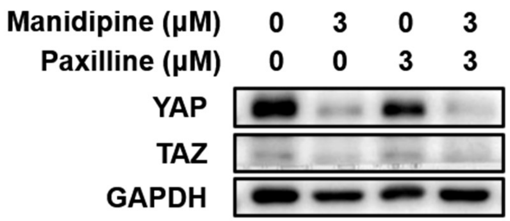

Figure S5. Effects of calcium and potassium channel blockers on sphere cell viability and YAP/TAZ expression in H460-SP cells.(a) Dose-response curves (left) and sphere cell viability (right) of H460-SP treated with the voltage-gated calcium channel blocker manidipine (top; alone or in combination with 500 nM paxilline) or the BK potassium channel blocker paxilline (bottom; alone or in combination with 500 nM manidipine) for 6 days. GI50 values were calculated using non-linear regression analysis in GraphPad Prism 8 (GI50 = 2.321 µM for manidipine, GI50 = 6.145 µM for paxilline).(b) Western blot analysis of YAP and TAZ protein levels in H460-SP spheres treated with manidipine (3 µM) and paxilline (3 µM), alone or in combination, for 72 h. GAPDH was used as an internal loading control. Data are presented as the mean ± SD of three independent experiments (n = 3). Statistical significance was determined by one-way ANOVA followed by Tukey's post hoc test (*p < 0.05, **p < 0.01, ***p < 0.001, ****p < 0.0001 vs. control).

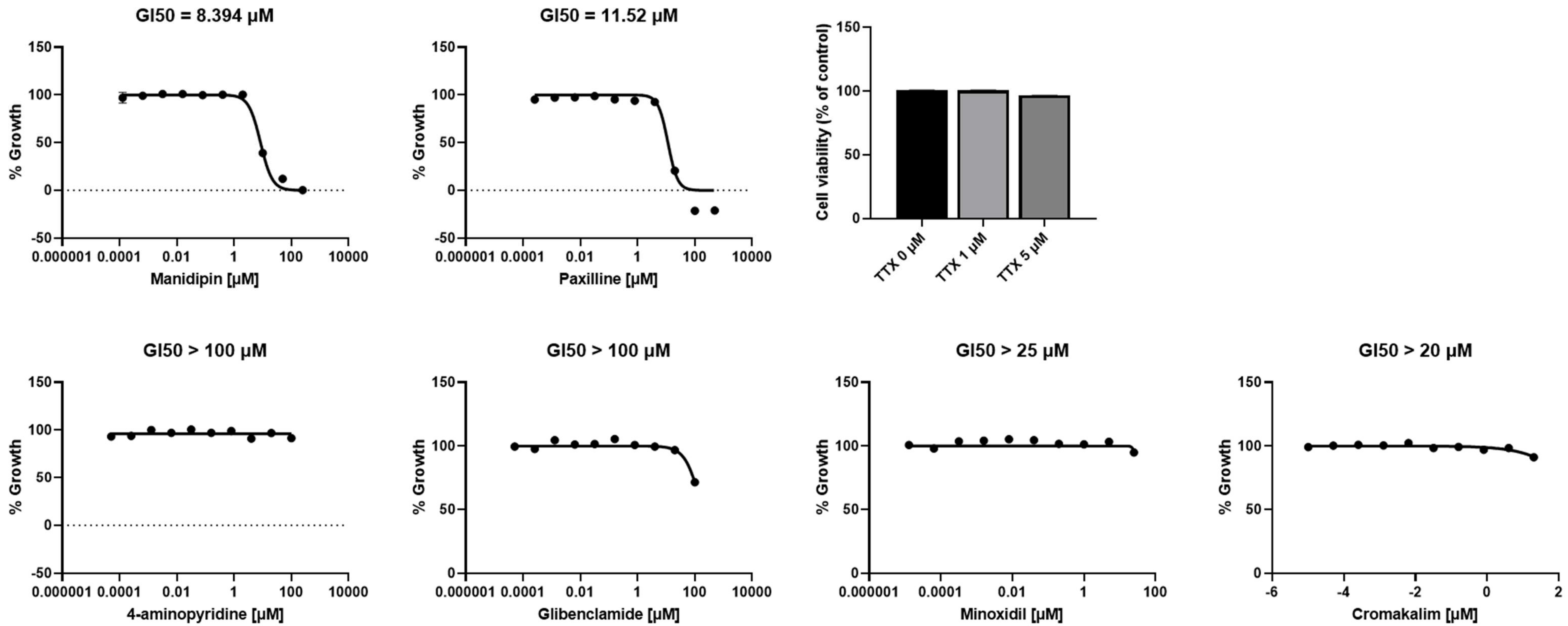

Figure S6. Cytotoxicity evaluation of various ion channel modulators in parental 2D H460 cells.Dose-response cell viability curves, bar graphs, and calculated GI50 values of parental 2D H460 lung cancer cells treated with various ion channel modulators for 72 h, measured using an ATP-based CellTiter-Glo assay. Compounds tested include the calcium channel blocker manidipine (GI50 = 8.394 µM), potassium channel blockers/modulators paxilline (GI50 = 11.52 µM), 4-aminopyridine (GI50 > 100 µM), glibenclamide (GI50 > 100 µM), minoxidil (GI50 > 25 µM), and (GI50 > 20 µM), as well as the voltage-gated sodium channel blocker tetrodotoxin (TTX; 1 and 5 µM). GI50 values were determined by non-linear regression analysis using GraphPad Prism 8. Data are presented as the mean ± SD of three independent experiments (n = 3).

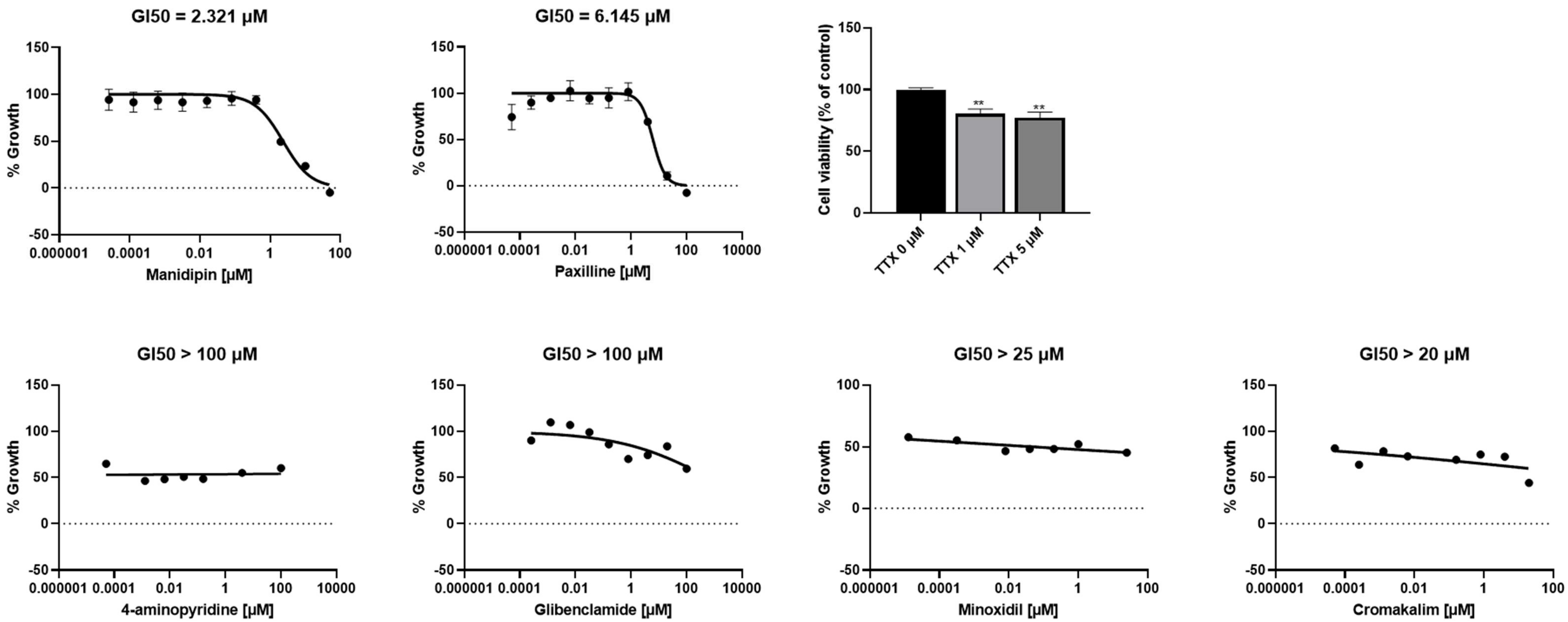

Figure S7. Cytotoxicity evaluation of various ion channel modulators in 3D H460-SP. Dose-response sphere cell viability curves, bar graphs, and calculated GI50 values of H460-SP treated with various ion channel modulators for 6 days, measured using an ATP-based CellTiter-Glo 3D assay. Compounds tested include the voltage-gated calcium channel blocker manidipine (GI50 = 2.321 µM), potassium channel blockers/modulators paxilline (GI50 = 6.145 µM), 4-aminopyridine (GI50 > 100 µM), glibenclamide (GI50 > 100 µM), minoxidil (GI50 > 25 µM), and (GI50 > 20 µM), as well as the voltage-gated sodium channel blocker tetrodotoxin (TTX; 1 and 5 µM). GI50 values were determined by non-linear regression analysis using GraphPad Prism 8. Data are presented as the mean ± SD of three independent experiments (n = 3).

Organoid Model	KCLB No.	Origin	Species / Age / Sex	Histopathology	Growth Pattern
SNU-2627-CO	02627-CO	Lung	Human / 46 / Female	Non-small cell lung cancer (NSCLC)	Compact organoid
SNU-2693-CO	02693-CO	Lung	Human / 60 / Male	Adenocarcinoma	Spheroidal, asymmetric, co mpact, luminal

Table S2. Clinicopathological characteristics and growth characteristics of patient-derived organoid (PDO) models.

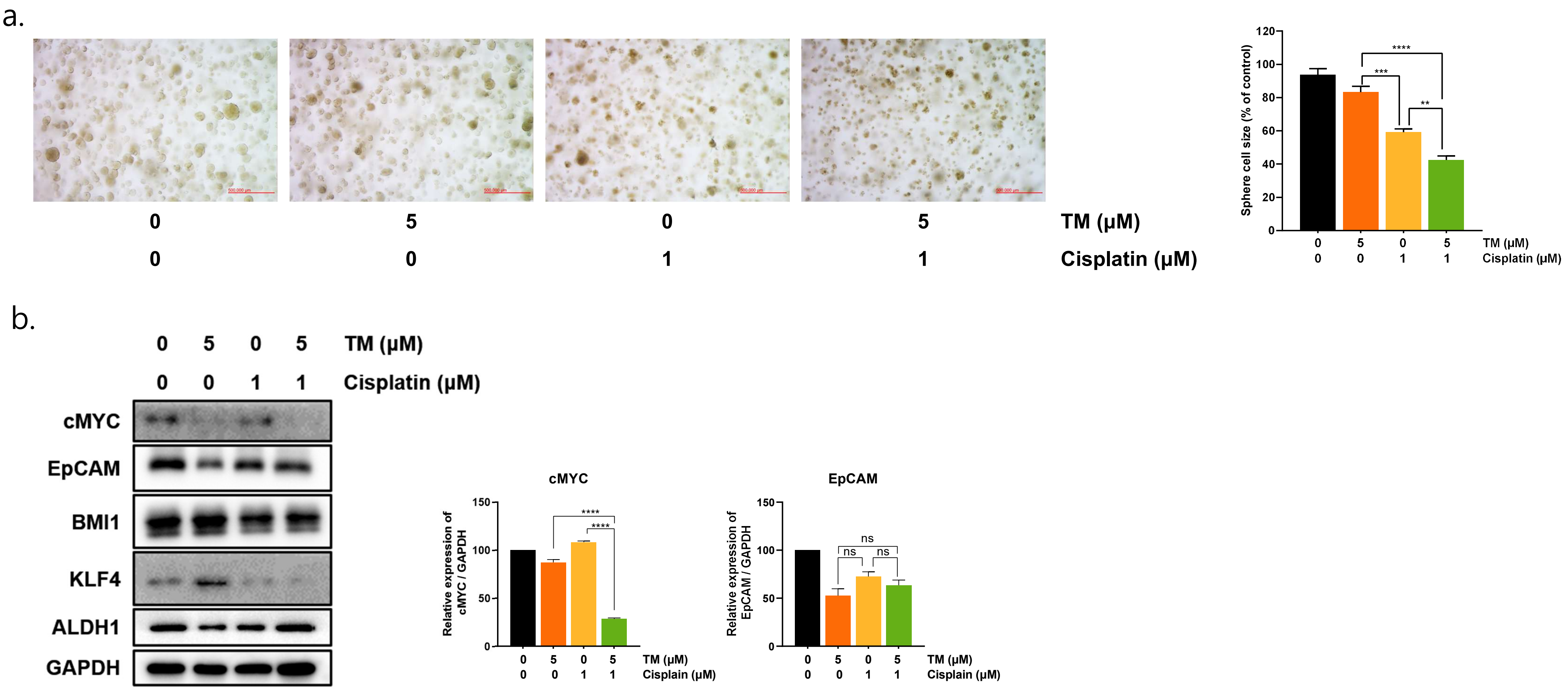

Figure S8. Combination effect of trimebutine and cisplatin on stemness and organoid size in patient-derived tumor organoids (SNU-2693-CO).(a) Representative brightfield images (left; scale bar = 500μm) and quantitative analysis of relative organoid size (right) of SNU-2693-CO lung adenocarcinoma patient-derived organoids (PDOs) treated with vehicle control, trimebutine (5μM), cisplatin (1μM), or their combination for 72 h.(b) Western blot analysis of stemness markers (cMYC, EpCAM, BMI1, KLF4, and ALDH1) in SNU-2693-CO PDOs treated with trimebutine (5μM) and/or cisplatin (1μM) for 72 h. Representative immunoblots (left) and quantitative densitometric analysis of cMYC and EpCAM protein levels normalized to GAPDH (right) are shown. Data are presented as the mean ± SD of three independent experiments (n=3). Statistical significance was determined by one-way ANOVA (∗p<0.05,p<0.01, ∗∗∗p<0.001, ∗∗∗∗p<0.0001; ns, not significant vs. control).**
